# Comparison of T2 values of the displaced unilateral disc and retrodiscal tissue of temporomandibular joints and their implications

**DOI:** 10.1038/s41598-024-52092-6

**Published:** 2024-01-19

**Authors:** Naoya Kakimoto, Pongsapak Wongratwanich, Hiroaki Shimamoto, Jira Kitisubkanchana, Tomomi Tsujimoto, Kiichi Shimabukuro, Rinus G. Verdonschot, Yoko Hasegawa, Shumei Murakami

**Affiliations:** 1 Department of Oral and Maxillofacial Radiology, Graduate School of Biomedical and Health Sciences, Hiroshima University, Hiroshima, Japan; 2 Division of Oral Diagnosis, Department of Oral Biomedical Sciences, Khon Kaen University, Khon Kaen, Thailand; 3 Department of Oral and Maxillofacial Radiology, Osaka University Graduate School of Dentistry, Osaka, Japan; 4 Department of Oral and Maxillofacial Radiology, Faculty of Dentistry, Mahidol University, Bangkok, Thailand; 5 Department of Oral and Maxillofacial Radiology, Hiroshima University Hospital, Hiroshima, Japan; 6 Max Planck Institute for Psycholinguistics, Nijmegen, The Netherlands; 7 Division of Comprehensive Prosthodontics, Niigata University Graduate School of Medical and Dental Sciences, Niigata, Japan

**Keywords:** Diseases, Oral diseases, Musculoskeletal system, Musculoskeletal system, Clinical trial design

## Abstract

Unilateral anterior disc displacement (uADD) has been shown to affect the contralateral joints qualitatively. This study aims to assess the quantitative T2 values of the articular disc and retrodiscal tissue of patients with uADD at 1.5 Tesla (T). The study included 65 uADD patients and 17 volunteers. The regions of interest on T2 maps were evaluated. The affected joints demonstrated significantly higher articular disc T2 values (31.5 ± 3.8 ms) than those of the unaffected joints (28.9 ± 4.5 ms) (*P* < 0.001). For retrodiscal tissue, T2 values of the unaffected (37.8 ± 5.8 ms) and affected joints (41.6 ± 7.1 ms) were significantly longer than those of normal volunteers (34.4 ± 3.2 ms) (*P* < 0.001). Furthermore, uADD without reduction (WOR) joints (43.3 ± 6.8 ms) showed statistically higher T2 values than the unaffected joints of both uADD with reduction (WR) (33.9 ± 3.8 ms) and uADDWOR (38.9 ± 5.8 ms), and the affected joints of uADDWR (35.8 ± 4.4 ms). The mean T2 value of the unaffected joints of uADDWOR was significantly longer than that of healthy volunteers (*P* < 0.001). These results provided quantitative evidence for the influence of the affected joints on the contralateral joints.

## Introduction

Temporomandibular joint (TMJ) disc displacement is one of the most common temporomandibular disorders (TMD)^1^, with a high prevalence ranging from 65 to 89% in symptomatic patients^2–6^, and from 25 to 31%^4–6^ in asymptomatic volunteers. Bilateral disc displacement affects 47.3–72.5% of symptomatic patients^3,4, 7, 8^, while unilateral disc displacement occurs in only 21–52.7%^3,4, 7^. The TMJ is bilaterally attached to the mandible and operates harmoniously as a single unit. According to several reports, unilateral anterior disc displacement (uADD) can significantly alter the overall behavior of the contralateral joint^9,10^, and has also been shown to cause skeletal asymmetry^11,12^. Another study discovered that the articular disc on the pain-free side of uADD patients exhibited up to 60% nonreducing displacement, and more than half of these joints experienced discomfort within two years after an episode of contralateral symptoms^13^. These findings highlight the necessity of using advanced imaging modalities to identify potential influence on the contralateral joint in patients with unilateral disc displacement. However, routine morphologic magnetic resonance (MR) imaging cannot demonstrate this early phenomenon. Therefore, biochemical imaging or T2 mapping is adopted to explore the changes through the T2 value (or T2 relaxation time), a quantitative MR parameter obtained with multi-echo spin-echo sequences. T2 mapping reveals changes in water and collagen content, reflecting degenerative processes indirectly. Any disruption to either the content or alignment of collagen fibers can increase the T2 values^14,15^ as the water molecule becomes more mobile or freely moving. Several studies have introduced the application and feasibility of T2 mapping for TMJ imaging^16–18^.

Furthermore, research has revealed that patients with anterior disc displacement without reduction and patients with TMD have longer T2 values of the articular disc and retrodiscal tissue than in asymptomatic volunteers^19,20^. Despite these advances, there remains a knowledge gap. To the best of our knowledge, no study has compared T2 values between the unaffected and affected sides of the articular disc and retrodiscal in patients with unilateral anterior disc displacement.

The primary objective of this study is to explore the impact of affected joints on their unaffected contralateral counterparts by assessing and comparing quantitative T2 values in both the articular disc and retrodiscal tissue among patients with uADD and healthy volunteers. This study aims to further evaluate the subgroups of uADD with reduction (WR) and without reduction (WOR), and within uADD subgroups based on the presence or absence of joint effusion, osteoarthritis, and bone edema.

## Results

### Patients’ characteristics

Sixty-five uADD patients were enrolled in this study based on the diagnosis from MR images (55 females and 10 males; median age, 43 years; age range, 14–77 years), comprising 15 uADDWR and 50 uADDWOR patients. Out of 65 patients with uADD, only eight did not show any signs of joint effusion (JE), osteoarthritis (OA), and bone marrow edema and/or osteonecrosis (BE) findings (wo JE OA BE). For those 17 healthy volunteers (five females and 12 males; median age, 26 years; age range, 23–32 years), MRI was also used to verify normal superior (NorSup) disc position (see Table 1).

**Table 1:** Patient and volunteer characteristics.

Variables	n (%)
Patients (cases)	65
Male	10 (15)
Female	55 (85)
Age of patients (years)	
Mean	43.9 ± 18.8
Median	43
Range	14–77
Patients’ disc position and function (joints)	
uADDWR	15 (23)
uADDWOR	50 (77)
uADD w JE OA BE	57 (88)
uADD wo JE OA BE	8 (12)
Volunteers (cases)	17
Male	12 (71)
Female	5 (29)
Age of volunteers (years)	
Mean	26.9 ± 2.4
Median	26
Range	23–32

*uADDWR* unilateral anterior disc displacement with reduction, *uADDWOR* unilateral anterior disc displacement without reduction, *uADD w/wo JE OA BE* unilateral anterior disc displacement with/without joint effusion (JE), *OA* osteoarthritis, and bone marrow edema and/or osteonecrosis (BE) findings.

### Comparison between uADD patients (n = 65) and normal volunteers (n = 17)

**Figure 1:**
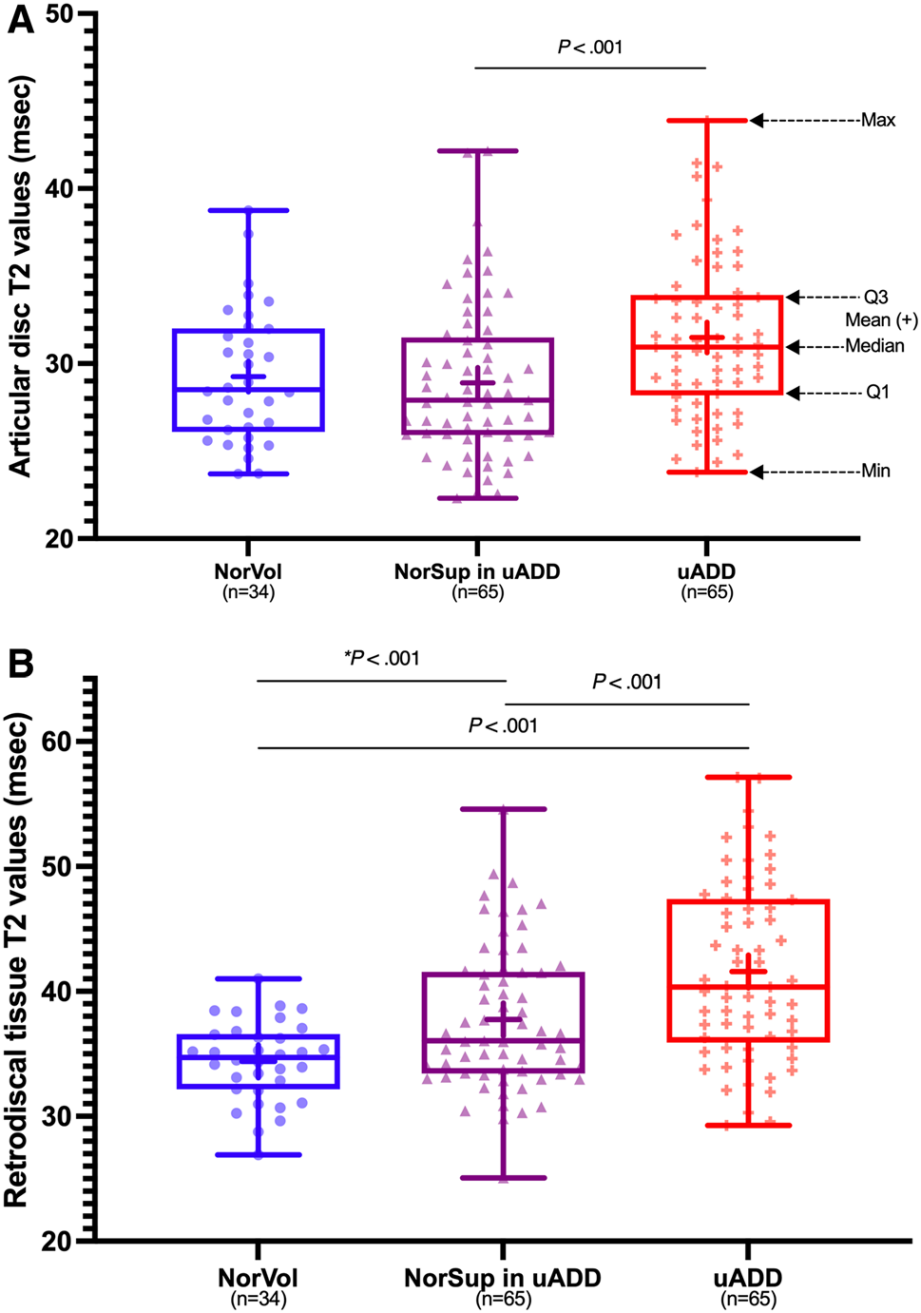
Box and whisker plots of T2 values derived from the regions of interest (ROIs). (**A**) shows the articular disc T2 values of normal volunteers (NorVol), unaffected joints (NorSup in uADD), and affected joints (uADD). (**B**) shows the retrodiscal tissue T2 values of NorVol, NorSup in uADD, and uADD. The group comparison between subgroups was performed using generalized estimation equations, and *P* values are presented above the box plots.

The mean T2 values of the articular disc were 29.3 ± 3.8 ms for normal volunteers, 28.9 ± 4.5 ms on the unaffected side, and 31.5 ± 3.8 ms on the affected side for uADD patients. The mean T2 value of the articular disc in affected TMJs of uADD patients was significantly longer than that of the unaffected (*P* < 0.001) and healthy (*P* < 0.014) TMJs (Fig. 1A). No significant difference was observed between unaffected and healthy joints (*P* < 0.695).

When adjusting for age and sex, the mean T2 value on the affected side remained significantly longer than that on the unaffected side (*P* < 0.001). However, the difference between the affected side and healthy volunteers was no longer statistically significant (*P* = 0.393).

For retrodiscal tissue, the mean T2 value of normal volunteers was 34.4 ± 3.2 ms. The unaffected and affected sides of uADD patients had retrodiscal tissue T2 values of 37.8 ± 5.8 and 41.6 ± 7.1 ms, respectively. The affected TMJs demonstrated significantly longer retrodiscal tissue T2 values than those in the volunteers and unaffected TMJs (*P* < 0.001). Moreover, the mean T2 value of the retrodiscal tissue in the unaffected TMJs was also significantly longer than that of normal volunteers (*P* < 0.001) (Fig. 1B).

After adjusting for age and sex, the mean T2 values in the affected joints remained significantly longer than those in the unaffected sides (*P* < 0.001) and in the volunteers (*P* = 0.005). Meanwhile, comparisons between unaffected sides and normal volunteers were no longer statistically significant (*P* = 0.666).

### Comparison between uADD patients without joint effusion, osteoarthritis, and bone marrow edema and/or osteonecrosis findings (wo JE OA BE) (n = 8) and normal volunteers (n = 17)

**Figure 2:**
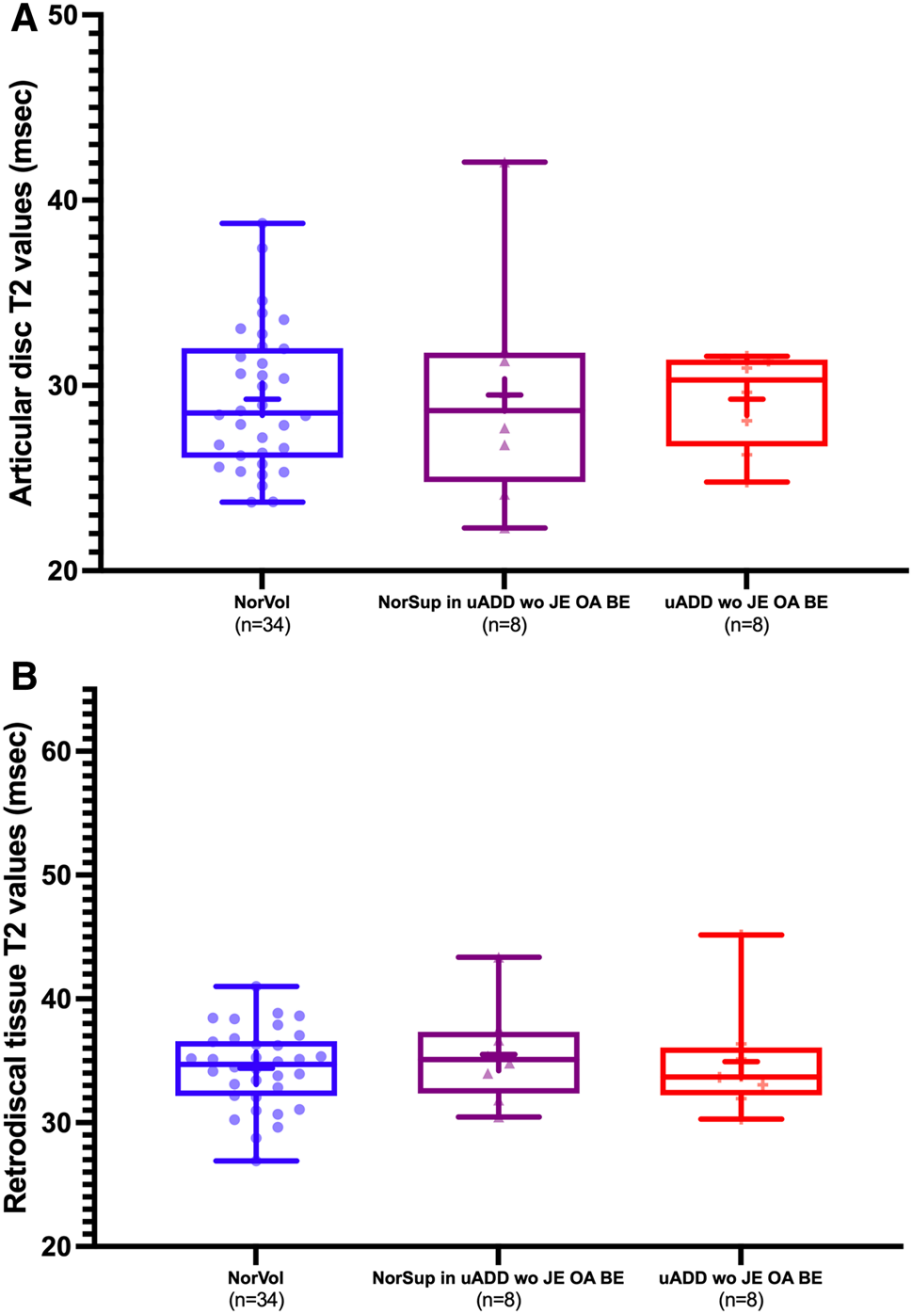
Box and whisker plots of T2 values derived from the regions of interest (ROIs). (**A**) shows the articular disc T2 values of normal volunteers (NorVol), unaffected joints (NorSup in uADD wo JE OA BE), and affected joints without joint effusion, osteoarthritis, and bone effusion (uADD wo JE OA BE). (**B**) shows the retrodiscal tissue T2 values of NorVol, NorSup in uADD wo JE OA BE, and uADD wo JE OA BE. No differences were observed in both plots (**A**, **B**).

There were no significant differences among the articular disc mean T2 values of normal volunteers (29.3 ± 3.8 ms), unaffected joints (29.5 ± 6.1 ms), and affected joints of uADD wo JE OA BE patients (29.3 ± 2.6 ms) (normal volunteers versus unaffected joints, *P* = 0.918; normal volunteers versus affected joints, *P* = 0.997; unaffected versus affected joints, *P* = 0.917) (Fig. 2A).

Likewise, for the mean T2 values of retrodiscal tissue, no differences were found among normal volunteers (34.4 ± 3.2 ms), unaffected joints (35.5 ± 3.9 ms), and affected joints of uADD wo JE OA BE patients (34.9 ± 4.5 ms) (normal volunteers versus unaffected joints, *P* = 0.465; normal volunteers versus affected joints, *P* = 0.752; unaffected versus affected joints, *P* = 0.754)) (Fig. 2B). After incorporating age and sex into the analysis, all results remained the same for both the articular disc and retrodiscal tissue.

### Comparison of uADDWR patients (n = 15), uADDWOR patients (n = 50), and normal volunteers (n = 17)

**Figure 3:**
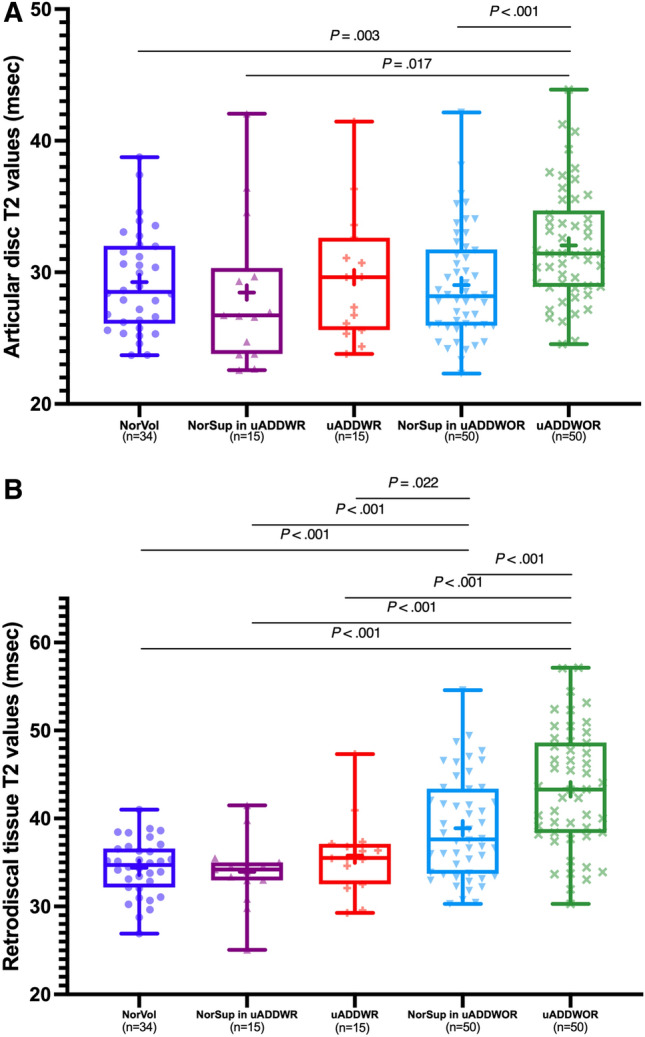
Box and whisker plots of T2 values derived from the regions of interest (ROIs). (**A**) shows the articular disc T2 values of normal volunteers (NorVol), unaffected joints of patients with disc displacement *with* reduction (NorSup in uADDWR), affected joints of patients with disc displacement *with* reduction (uADDWR), unaffected joints of patients with disc displacement *without* reduction (NorSup in uADDWOR), and affected joints of patients with disc displacement *without* reduction (uADDWOR) (**B**) shows the retrodiscal tissue T2 values of NorVol, NorSup in uADDWR, uADDWR, NorSup in uADDWOR, and uADDWOR. The comparison between subgroups was performed using generalized estimation equations, and *P*-values are presented above the box plots.

The mean T2 values of the articular disc were 29.3 ± 3.8 ms in normal volunteers, 28.5 ± 5.5 ms in the unaffected side of uADDWR, and 29.6 ± 4.9 ms in the affected sides of uADDWR patients. For patients with uADDWOR, the mean T2 values of the articular disc were 29.0 ± 4.1 ms in unaffected TMJs and 32.0 ± 4.4 ms in affected TMJs. The affected joints of uADDWOR patients had significantly longer T2 values of the articular disc than those in the healthy (*P* = 0.003) and unaffected TMJs of both uADDWR (*P* = 0.017) and uADDWOR (*P* < 0.001) patients (Fig. 3A). However, no differences were observed between the affected joints of uADDWR and uADDWOR patients (*P* = 0.076).

After controlling for age and sex, only the difference between the affected and unaffected sides of uADDWOR remained statistically significant (*P* < 0.001). When comparing the affected joints of uADDWOR patients with healthy volunteers (*P* = 0.251) and the unaffected TMJs of uADDWR (*P* = 0.097), the differences were no longer significant.

For retrodiscal tissue, the mean T2 values of normal volunteers were 34.4 ± 3.2 ms. Unaffected and affected joints of uADDWR patients had T2 values of 33.9 ± 3.8 and 35.8 ± 4.4 ms, respectively. Patients with uADDWOR had retrodiscal tissue T2 values of 38.9 ± 5.8 ms in the unaffected TMJs and 43.3 ± 6.8 ms in the affected TMJs. The mean T2 value of the retrodiscal tissue in the affected side of uADDWOR patients was significantly longer than that of normal volunteers (*P* < 0.001), both the affected (*P* < 0.001) and unaffected joints of uADDWR patients (*P* < 0.001) and unaffected joints of uADDWOR patients (*P* < 0.001). Moreover, the mean T2 value of the retrodiscal tissue in the unaffected joints of uADDWOR patients was significantly longer than that in normal volunteers (*P* < 0.001) and in unaffected joints of uADDWR patients (*P* < 0.001) (Fig. 3B).

After considering age and gender as confounders, all results remained the same, except that the difference between unaffected joints of uADDWOR patients and normal volunteers was no longer significant (*P* = 0.558).

## Discussion

Bilateral TMJ disc displacement is a commonly reported occurrence^3,4, 7, 8^. Evidence suggests that asymptomatic joints with displaced discs are more likely to develop pain in patients experiencing unilateral symptoms^13^. Consequently, it is plausible that uADD could have an effect on healthy contralateral joints. This study is the first to show that the T2 values of both the articular disc and retrodiscal tissue of the affected joints were longer than those of the unaffected joints. We also discovered that the unaffected joints had a more extended T2 value of retrodiscal tissue than normal volunteers. This finding supports the idea that the right and left TMJs work uniformly, with one likely influencing the other. This is consistent with previous reports that unilaterally displaced discs can have an impact on contralateral joints^9,10^. A current study, however, did not observe such a tendency for the articular disc, as there was no difference in the articular disc T2 value of volunteers and unaffected joints of patients with uADD. It could be due to the fact that retrodiscal tissue is highly vascularized, easily stretches, and loses its elasticity, making it more sensitive to changes in the fluid content, resulting in longer T2 values. Furthermore, changes in retrodiscal tissue typically occur as a compensatory mechanism for anterior disc movement before the permanent development of ADD, suggesting that an increase in T2 values may precede changes in the disc. In contrast, the articular disc is densely packed with collagenous fibers without any blood supply and may be less water-sensitive compared to retrodiscal tissue; as a result, early degenerative changes may take longer to manifest. Given the potential impact of underpowering on our findings, the possibility that a larger sample size might reveal subtle yet significant differences in T2 values in the articular disc should be recognized. Other factors, such as asymmetry and anatomical variations in mandibular shape and position, as well as TMJ biomechanics, should be taken into account because they may also play a role in the TMD predisposition of unaffected discs.

A comparison between the uADD wo JE OA BE patients and normal volunteers revealed no significant differences in the T2 values for both the articular disc and retrodiscal tissue. Our findings suggest that the sole impact of ADD on T2 values, in the absence of other defects, is perhaps not strong. In line with our observations, a study by Bristela et al. found no significant difference in the T2 values regarding the disc position or MR signal intensity; however, once the effect of signal intensity was considered together with disc position, a statistical difference in T2 value emerged^22^. However, drawing conclusions about the little impact of uADD wo JE OA BE on T2 values can be challenging due to the relatively small number of this subgroup and these presentations (JE OA BE) are not independent of each other, with one potentially influencing the other.

Moreover, demographic features such as age and sex could be confounding variables affecting T2 values. However, after accounting for these factors through multivariable analyses, the results of comparing uADD wo JE OA BE patients and volunteers remained the same. In contrast, other subgroup comparisons (uADD versus normal volunteers and uADDWR versus uADDWOR versus normal volunteers) yielded slightly different results, highlighting the influence of age and sex on the T2 values.

According to our published studies, the T2 values of both the articular disc and retrodiscal tissue in patients with signs of joint effusion, osteoarthritis, and bone marrow abnormalities were reported to be longer than those in patients who did not portray these signs^19,20^. To date, several studies have reported that the T2 values of larger joints (knees) are susceptible to effects from surrounding structures such as joint effusion^22,23^, and can potentially be even greater in smaller joints, namely the TMJ. From the above, we suggest that MR features, besides disc displacement, could significantly impact T2 values. These might explain why no differences between uADD wo JE OA BE patients and normal volunteers were observed.

We also investigated the T2 values in greater detail among normal volunteers, uADDWR, and uADDWOR patients. The investigation showed that the affected joints of uADDWOR patients had significantly longer T2 values of both the articular disc and retrodiscal tissue than the unaffected joints regardless of the reduction status, normal volunteers, and affected joints of uADDWR patients (only the value of retrodiscal tissue). Additionally, the retrodiscal tissue T2 value of the unaffected side of the uADDWOR patients was significantly longer than that of the volunteers and the unaffected side of uADDWR patients. These findings are in agreement with a previous study reporting the increased signal intensity of the posterior band in the following order from low to high: normal, posterior DDWR, ADDWR, and ADDWOR^24^. In addition, many studies have established a relationship between ADDWOR and degenerative lesions, showing a higher prevalence of disc deformation^25^, joint effusion^26–28^, and osteoarthrosis^29^, which could explain the increase in T2 values.

This study did have some limitations. The first limitation was the scanning protocol. The unadjustable default protocol from the provider included eight echo times. It is important to note that a higher number of echo times has the potential to decrease the overall signal-to-noise ratio (SNR). Moreover, a 1.5T MR machine was used at the time of conducting this research due to limited resources. Higher-field strength scanners (e.g., 3.0T) have been suggested for TMJ evaluation^30^ as they improve the structural analysis^31^ and the perceptibility of small joints^32^. In addition, the 3.0T MR provides more signal availability, allowing the acquisition matrix to be increased to produce images with smaller voxels. SNR will double from 1.5T MR if the acquisition matrix remains constant; in other words, a better TMJ definition can be achieved^33^. However, some artifacts, such as noise, metal and motion artifacts, can also be greater under a stronger magnetic field.

Another limitation was the age and gender imbalance in our study population, with the uADD patients having an older age profile and with a higher proportion of females in the affected group and more males in the healthy group. Consequently, these may introduce a potential bias in our results. It is essential to consider this imbalance when interpreting the study’s outcomes. We suggest further studies to adopt an age-matched approach and strive for a more balanced gender distribution. These adjustments will ensure a more comprehensive representation of the population of interest.

Additionally, it is essential to note that the study was cross-sectional, meaning that the T2 values could be influenced by a variety of factors that change over time, such as natural progression of the disease, interventions received, or other external influences. As a result, the study’s ability to predict the effect on the T2 values is limited. Moreover, our study did not collect data on the onset and duration of the disease in participants. This lack of data prevents us from conducting analyses of the disease chronicity, which could be a covariate in determining T2 values. Our findings, on the other hand, validate the associations observed during the specific timeframe of the study.

For the first time, this study has demonstrated the association between biochemical changes on the unaffected TMJ in patients with uADD, possibly arising from the influence of the diseased joint on the opposite side. This study also provides a comparative analysis of the quantitative T2 values among patients with unilateral disc displacement and normal volunteers. We confirmed the importance of this influence on the contralateral joints in patients with uADD. We recommend using the T2 mapping as a tool to capture the biochemical alterations inside the unaffected TMJ, which conventional imaging is incapable of.

## Methods

### Study population

This cross-sectional study was approved by the Institutional Review Board of Osaka University Graduate School of Dentistry (H21-E16). The procedures used in this study were in accordance with the Declaration of Helsinki. Informed consent was obtained from all the participants and/or their legal guardians after explaining the nature of the procedures.

**Figure 4:**
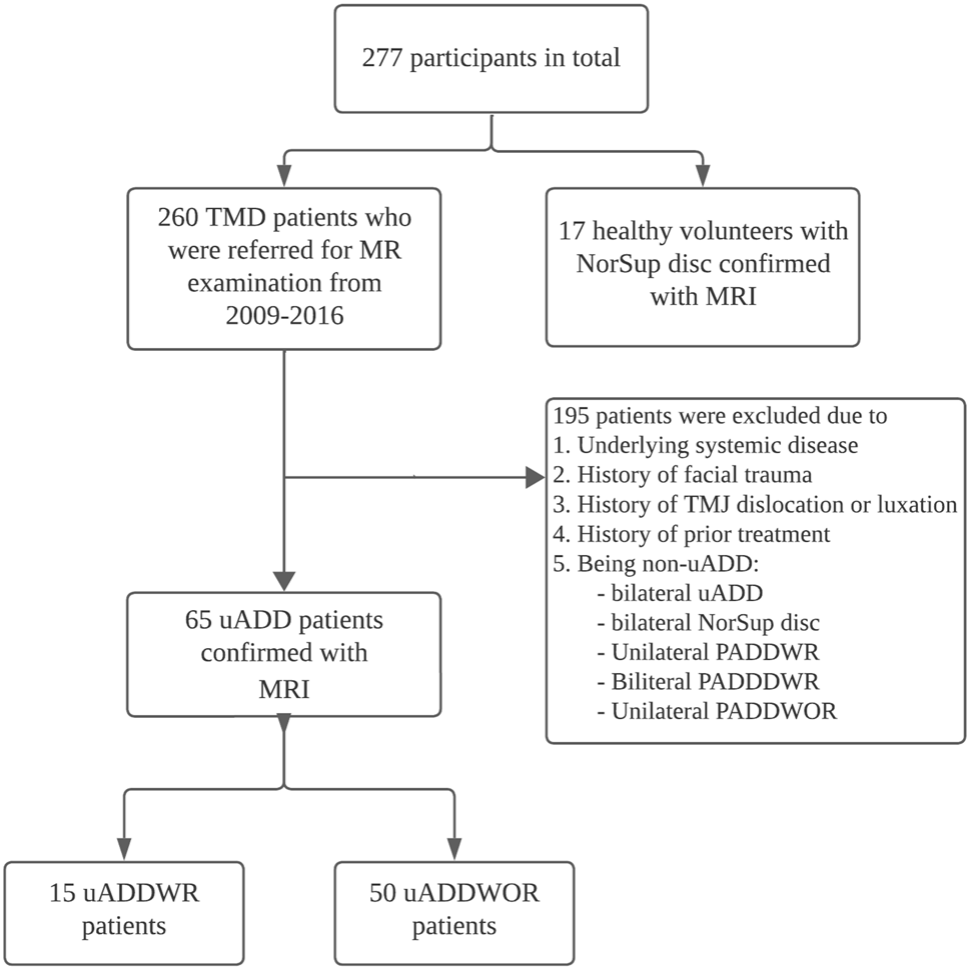
Patient selection diagram. TMD = temporomandibular disorder, NorSup = normal superior, uADD = unilateral anterior disc displacement, uADDWR = unilateral anterior disc displacement with reduction, uADDWOR = unilateral anterior disc displacement without reduction, PADDWR = partial anterior disc displacement with reduction, PADDWOR = partial anterior disc displacement without reduction.

Between 2009 and 2016, 260 consecutive patients clinically diagnosed with TMD (as shown in Fig. 4) including TMJ or orofacial pain, mandibular dysfunction (clicking, crepitation, locking), or signs suggestive of internal derangement. Prior to starting any treatment, the patients were referred to the Osaka University Dental Hospital to receive an MR examination with T2 mapping sequences. Patients with the following conditions were excluded from the study: (1) presence of any underlying systemic diseases, (2) history of facial trauma, (3) history of TMJ dislocation or subluxation, (4) history of prior treatments, and (5) non-uADD. Following MRI confirmation of uADD, 65 uADD patients (uADDWR (n = 15) and uADDWOR (n = 50)) were included in the study. In addition, 17 healthy volunteers with no indications for an MR examination were recruited, based on convenience sampling, as the control group (a total of 34 joints). Each volunteer had bilateral normal articular disc positions confirmed by MRI.

### MR examination

All subjects were examined using a 1.5T MR scanner (Signa HDxt 1.5T; GE Healthcare, Milwaukee, Wisconsin, USA) equipped with a TMJ surface coil. The imaging protocol (see Table 2) for TMD diagnosis included oblique sagittal and coronal fast spin-echo proton density-weighted imaging (FSE-PDWI) and fat-suppressed fast spin-echo T2 weighted image (FSE-T2WI) sequences, perpendicular and parallel to the long axis of the mandibular condyle in a closed mouth position. In addition, the sagittal FSE-PDWI sequences were obtained in both closed and open-mouth positions. To measure T2 values, T2 mappings (oblique sagittal FSE sequences) were performed in a closed mouth position at eight different echo times as follows: 8.9, 17.8, 26.7, 35.6, 44.5, 53.4, 62.4, 71.3 ms. For the T2 mapping sequence, the total acquisition time of each joint was 5 min and 22 s, as previously described^19,20^.

**Table 2:** Scan parameters.

	FSE-PDWI oblique sagittal and coronal	Fat-suppressed FSE-T2WI sagittal	FSE-PDWI sagittal open mouth position	T2 mapping at oblique sagittal
FOV (mm × mm)	120 × 120	120 × 120	120 × 120	120 × 120
Acquisition matrix	256 × 160	256 × 160	256 × 160	256 × 160
Slice thickness (mm)	3	3	3	4
Slice gap (mm)	1	1	1	1
TR (ms)	2500	2000	800	1000
TE (ms)	20	85	24	8.9–71.3
ETL	8	16	4	8
NEX	2	3	2	2

*FOV* a field of view, *TR* repetition time, *TE* echo time, *ETL* echo train length, *FSE-PDWI* fast spin-echo proton density-weighted image, *FSE-T2WI* fast spin-echo T2-weighted image, *NEX* number of excitations.

### MR image evaluation

All the MR images were independently assessed by two experienced oral and maxillofacial radiologists (N.K. with 20 years of experience and H.S. with 12 years of experience), blinded to all patient information. In cases of disagreement regarding diagnosis, a final consensus was reached. The morphological features evaluated were disc position, joint effusion, osteoarthritis, and bone marrow abnormalities^19^.

**Figure 5:**
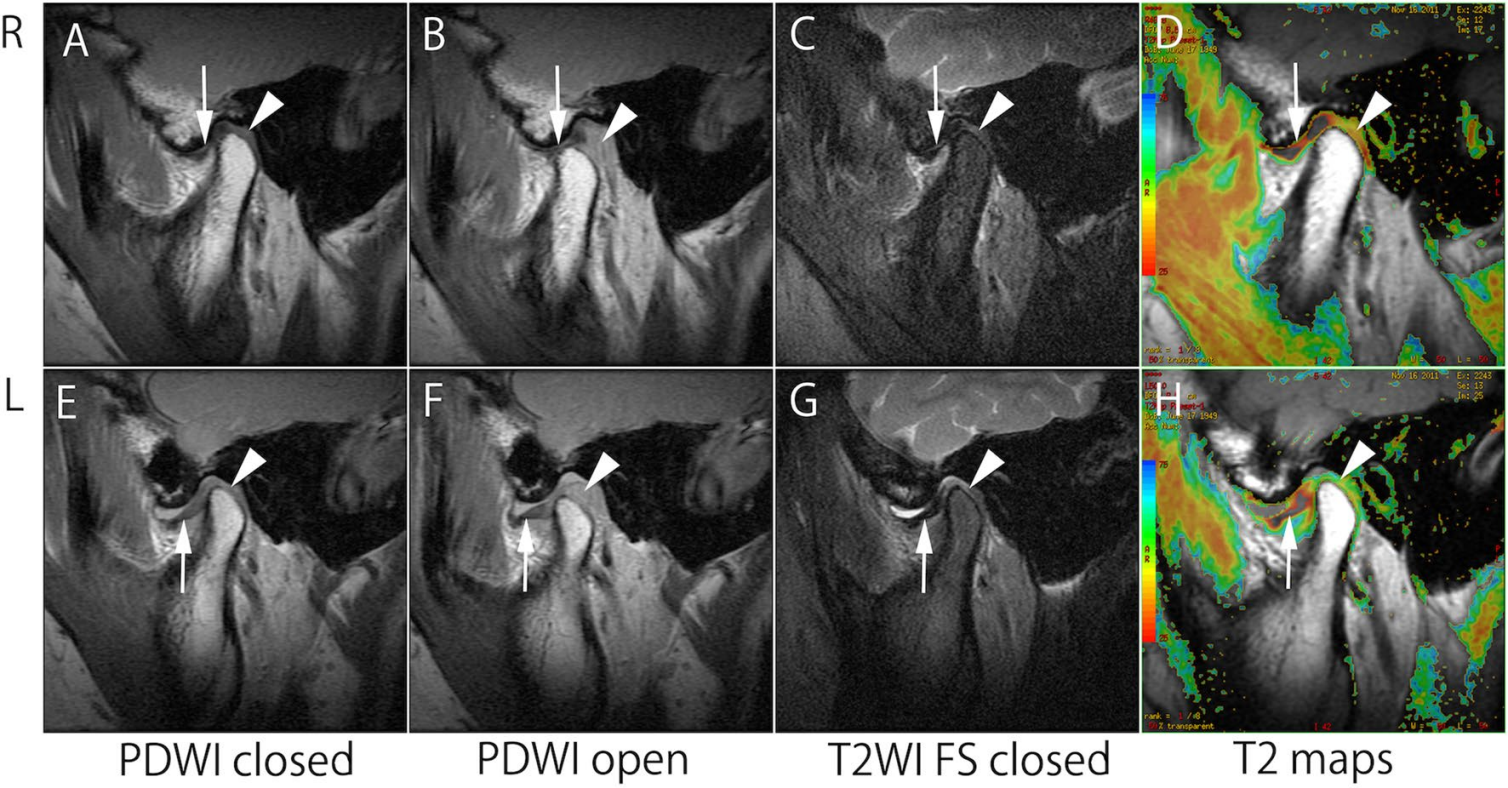
A 62-year-old female patient with unilateral ADDWOR and trismus. Right TMJ MR images (**A**–**D**) show normal superior disc position (NorSup) with normal function. The T2 values of the normal side were 25.8 ms for the articular disc (arrow) and 40.0 ms for retrodiscal tissue (arrowhead). Left TMJ MR images (**E**–**H**) show anterior disc displacement without reduction (ADDWOR) with joint effusion. The T2 values of the ADDWOR side were 29.1 ms for the articular disc and 51.0 ms for retrodiscal tissue.

Articular disc displacements were classified into five categories, as reported by Tasaki et al., with some minor modifications^4^. On oblique sagittal and sagittal FSE-PDWI in closed- and open-mouth positions, the possible classifications were normal superior (NorSup) (Fig. 5A and B), partial anterior disc displacement with reduction (PADDWR), partial anterior disc displacement without reduction (PADDWOR), anterior disc displacement with reduction (ADDWR), and anterior disc displacement without reduction (ADDWOR) (Fig. 5E and F). However, patients with PADDWR and PADDWOR were excluded from this study to decrease diagnostic ambiguity. Therefore, patients with a NorSup disc on one side (unaffected) with an ADDWR or ADDWOR disc on the other side (affected) were recruited and defined as uADD patients.

According to Larheim et al., the joint effusion was classified into four categories: none or minimal fluid (Fig. 5C), moderate, marked, and extensive fluid (Fig. 5G) on oblique sagittal fat-suppressed T2WIs in a closed mouth position^34^. As suggested by Kirk, osteoarthritis was graded as negative or positive for osteophytes or erosion^35^. When bone marrow edema and/or osteonecrosis were present on oblique sagittal FSE-PDWIs and T2WIs in a closed mouth position, the joint was classified as positive for bone marrow abnormalities, as defined by Larheim et al.^36^.

### Measurement of T2 values

Eight-echo FSE images were initially transferred to an independent workstation (Advantage Workstation ver. 4.4; GE Healthcare, Milwaukee, WI, USA). To measure the T2 value, two independent observers (N.K. and H.S.) manually and independently selected a total of four regions of interest (ROIs) based on anatomical location observed on proton-density weight images as previously conducted in past researches, including one ROI from the articular disc (with a coefficient of variation (CV%) ranging from 1.1 to 4.7% for intra-rater reliability and an intraclass correlation coefficient (ICC) of 0.862 in healthy volunteers and 0.891 in patients for inter-rater reliability)^18^ and the other three ROIs from the retrodiscal tissue including bilaminar zone, superior, and inferior attachment (with a CV% ranging from 1.5% to 5.5% for intra-rater reliability and an ICC of 0.818 in both healthy volunteers and patients for inter-rater reliability)^19,20^. T2 values were calculated using the Functool software (Functool 4.4.5, GE Healthcare, Milwaukee, Wisconsin, USA). Finally, the average T2 value determined by independent observers was defined as the definitive T2 value (Fig. 5D and H).

### Statistical analysis

The data were analyzed using IBM SPSS Statistics (Version 28.0)^21^. To compare the T2 values among healthy subjects, unaffected and affected sides of uADD patients, a linear regression model using generalized estimating equations (GEEs) was performed, taking into account the correlated data within uADD patients. A multivariable analysis was also conducted to control for potential confounding effects by age and sex. A *P* value < 0.05 was considered as statistical significance.

## Conclusions

In conclusion, our study showed a potential influence on the contralateral healthy TMJ of patients with uADD using a quantitative T2 value. We found that the T2 value of the retrodiscal tissue on the unaffected sides of uADDWOR patients was significantly increased. Therefore, we suggest that dental practitioners (and radiologists) might best keep in mind not to overlook the condition of the contralateral TMJ in uADD patients. Our findings suggest that disc displacement had a lower impact on the T2 value than other conditions. Moreover, as expected, ADDWOR showed the highest T2 values among all the groups, previously shown to be associated with chronic and degenerative changes.

## Data Availability

The datasets used and analyzed during the current study are available from the corresponding author on reasonable request.
